# Collision tumor of the appendix incidentally discovered in a patient diagnosed with an adenocarcinoma of the colon: a case report

**DOI:** 10.11604/pamj.2022.43.161.33298

**Published:** 2022-11-29

**Authors:** Syrine Moussa, Salwa Nechi, Abir Chaabane, Karim Mfarrej, Amel Dougaz, Mehdi Bouassida, Slim Zribi, Hassen Touinsi, Emna Chelbi

**Affiliations:** 1 Department of Pathology, Med Taher Maamouri University Hospital, 8050, Nabeul, Tunisia,; 2 Department of Gastrointestinal Surgery, Med Taher Maamouri University Hospital, 8050, Nabeul, Tunisia

**Keywords:** Appendiceal neoplasm, collision tumor, low-grade appendiceal mucinous neoplasia, neuroendocrine tumor, case report

## Abstract

Collision tumor of the appendix is an extremely rare entity, defined as the coexistence of two independent tumors located in the same site without transitional changes. We describe in this report the case of a 75-year-old man who presented with an acute abdominal pain, nausea and vomiting. Physical examination revealed an abdominal distension with a hypogastric mass. Abdominal computed tomography showed colonic obstruction related to a sigmoid tumor. Therefore, a total colectomy was made. Macroscopic specimen examination showed a sigmoid tumor associated to a cystic dilatation of the appendix tip with mucoid content. Histological examination of the appendix showed the co-existence of two independent tumors located in the tip, without transitional changes: pTis low-grade appendiceal mucinous neoplasia and grade 1 neuroendocrine tumor. The latter was discovered incidentally during histological examination. We draw attention through our presentation to the importance of a thorough macroscopic and histological examination of the appendix.

## Introduction

Primary tumors of the appendix are infrequent representing 1-2% of appendectomies [1,2]. Low-grade appendiceal mucinous neoplasia (LAMN) and well differentiated neuroendocrine tumor (NET) are the most common lesions and are incidentally found in 0.6% and 0.3-0.9% of appendectomy specimens respectively [1,3]. Collision tumors of the appendix are extremely rare, defined as the coexistence of two independent tumors located in the same site without transitional changes, giving rise to difficulties in pathologic diagnosis, therapeutic management and prediction of prognosis [4]. Therefore, we present a case of a 75-year-old man who was diagnosed with tumor of the sigmoid and in whom a collision tumor of the appendix of a low-grade mucinous neoplasm and a well differentiated neuroendocrine tumor was incidentally discovered.

## Patient and observation

**Patient information:** a 75-year-old man without significant pathological history presented at the emergency room with a sudden onset of an acute abdominal pain, nausea and vomiting.

**Clinical findings:** physical examination revealed an abdominal distension with a painful hypogastric mass.

**Timeline of current episode:** May 2021: the patient presented at the emergency room of our institution for abdominal pain associated with nausea and vomiting. Therefore, abdominal computed tomography (CT) scan was made in urgency and the patient was referred to the operating room.

**Diagnostic assessment:** computed tomography scan of the abdomen showed colonic occlusion related to a sigmoid tumor. Therefore, a total colectomy was made.

**Figure 1:**
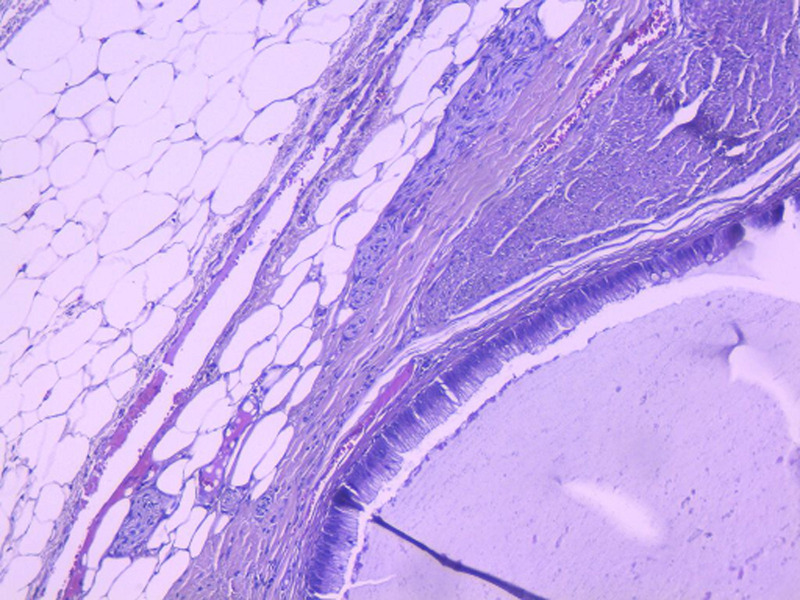
low-grade appendiceal mucinous neoplasia: cystic dilatation of the appendix tip lined by a well differentiated mucinous epithelium with flat and tubulous architecture associated with atrophy of the lymphoid tissue; the muscularis propria and the subserosa tissue were not infiltrated (HEx20)

**Figure 2:**
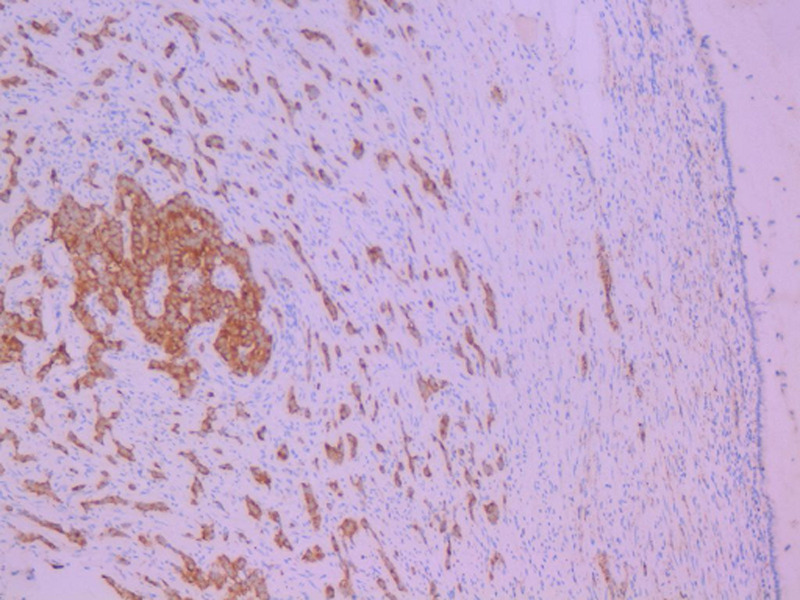
neuroendocrine tumor: granular cytoplasmic positivity to chromogranin (immunohistochemistry, x20)

**Figure 3:**
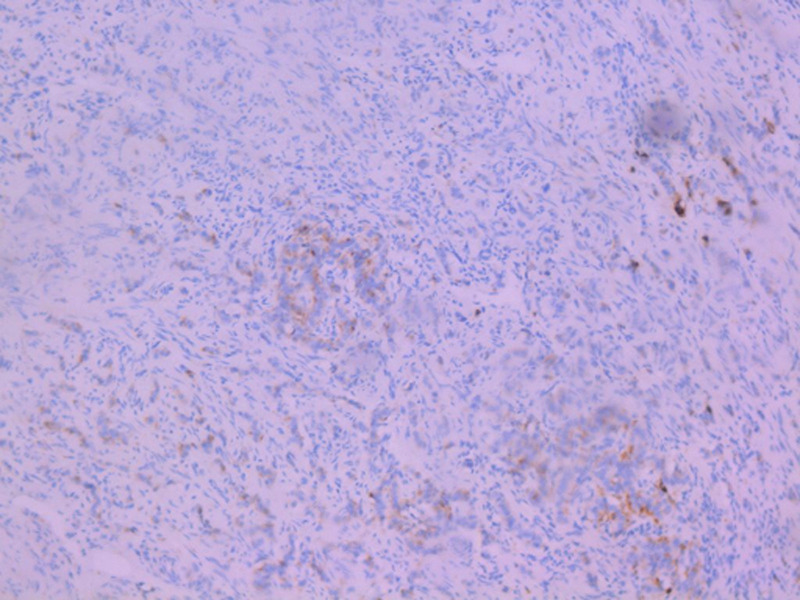
neuroendocrine tumor: granular cytoplasmic positivity to synaptophysin (immunohistochemistry, x20)

**Diagnosis:** macroscopic specimen examination objectified a stenosing tumor of the sigmoid, measuring 6cm and located 11cm from the colonic resection margin. We also noted a cystic dilatation of the appendix tip with mucoid content, which extends over 1.5cm. Elsewhere, the appendix was of conserved morphology. Therefore, the appendix was included in its entirety. The sigmoid tumor corresponded to an invasive low-grade adenocarcinoma which invaded through the serosa. Lympho-vascular space invasion was present and there was no lymph node metastasis. Appendix examination objectified the co-existence of two independent tumors located in the tip, without transitional changes: the first tumor consisted of a LAMN classified as pTis (Figure 1). The second tumor corresponded to a well differentiated, grade 1 NET measuring 0.3cm and it was incidentally found on histological examination. Resection margins were clear. There was no regional lymph node involvement and no mesoappendiceal invasion. The NET tumor cells displayed granular cytoplasmic positivity to chromogranin A and synaptophysin (Figure 2, Figure 3). The Ki-67 was less than 1% with no mitosis.

**Therapeutic interventions:** total colectomy.

**Follow-up and outcome of interventions:** the postoperative course was regular and the patient was discharged in good condition on the next postoperative day. The patient was then referred to the oncology department for possible adjuvant treatment.

**Patient perspective:** “I will continue with the adjuvant treatment in order to cure of the cancer”.

**Informed consent:** the patient expressed his absolute and informed consent.

## Discussion

Appendiceal tumors are uncommon entities accounting for 1-2% of all appendectomy specimens. They are usually found incidentally during appendectomy for appendicitis. Their clinical presentation is similar to acute appendicitis making the pre-operative diagnosis of appendiceal neoplasm challenging [1,2]. Collision tumor of the appendix is a rare entity defined as the coexistence of two independent neoplasms of biclonal origins, located in the same site without transitional changes [4]. Our review of the literature found a few published cases of appendiceal collision tumors involving LAMN and NET [1,4-8] summarized in Table 1.

**Table 1:** cases of collision tumor low-grade appendiceal mucinous neoplasia (LAMN) and neuroendocrine tumor (NET)

Author and year	Age (years)	Presentation	Histology	Treatment
Singh *et al*. (2011)	52	Abdominal discomfort + weight loss + pain and mass of the iliac fossa	Mucinous adenocarcinoma + NET	Right hemicolectomy + chemotherapy + excision of the omental deposit
Ekinci *et al*. (2018)	60	Abdominal discomfort + frequent pain in the right lower quadrant	LAMN + grade 1 NET	Appendectomy; NB: right hemicolectomy indicated but refused by the patient
Sholi *et al*. (2019)	23	Right lower quadrant pain + chronic constipation	LAMN + NET	Right hemicolectomy
Sugarbaker *et al*. (2020)	39	Lower quadrant and right-side pain	Ruptured LAMN + grade 1 NET	Right hemicolectomy + HIPEC
	32	Mucin discovered during inguinal hernia repair	Ruptured LAMN + grade 2 NET	Right hemicolectomy + omentectomy + cholecystectomy + HIPEC
Ruiz *et al*. (2021)	54	Acute appendicitis	LAMN + NET	Appendectomy
Villa *et al*. (2021)	31	Abdominal pain and dysuria	LAMN + grade 1 NET	Right hemicolectomy

LAMN: low grade appendiceal mucinous neoplasia; NET: neuroendocrine tumor; HIPEC: hyperthermic intraperitoneal chemotherapy

Singh *et al*. reported in 2011 a case of collision tumor of the appendix composed of an aggressive mucinous adenocarcinoma, with omental deposit and metastasis to the regional lymph nodes at the time of presentation, and a well differentiated NET [4]. Another case was described by Ruiz *et al*. in 2021; however, in this case the mucinous adenocarcinoma was confined to the appendix [8]. In our presentation, the patient was diagnosed with adenocarcinoma of the sigmoid; collision tumor of LAMN and NET was incidentally found in the appendix tip. A comparable case to our presentation was reported by Meeks *et al*. in 2016 describing a case of a 95-year-old woman with synchronous quadruple primary neoplasm. She was diagnosed with adenocarcinoma of the right colon, collision tumor of NET and Schwann cell hamartoma in the appendix and sessile serrated adenoma of the appendix. The collision tumor was discovered in the histological examination [9].

LAMNs are uncommon in the appendix, accounting for 0.6% of all appendiceal lesions. They can present as appendicitis, mucocele or as pseudomyxoma peritonei. LAMNs usually have a good prognosis if confined to the appendix. NETs are the most frequent tumors of the appendix and are found in 0.3-0.9% of appendectomy specimens with small predominance in female patients. Patients mostly present with appendicitis, carcinoid syndrome is rarely observed. NET rarely metastasize and they usually have an excellent prognosis [1,3].

The management of the appendiceal collision tumors remains a matter of debate. For LAMN, the assessment depends on the grade of atypia, lymph node involvement and peritoneal extension. Simple appendectomy is a sufficient treatment for LAMN, even when ruptured. However, this condition may cause recurrence by the development of pseudomyxoma peritonei. Thus, surgical debulking or post-operative intraperitoneal chemotherapy would be necessary [1-3]. The treatment of NETs is determined by tumor size and mitotic activity. For well differentiated NET < 1cm, appendectomy is usually curative. Right hemicolectomy should be indicated in all these cases: lesions > 2cm, lymph node involvement, high mitotic activity, mesoappendix spread and positive margins [3]. Surgical approach to LAMN and NET remains controversial. In fact, a recent studies conducted by Lamberti *et al*. and Gonzalez *et al*. found that there is no improved survival rates following right hemicolectomy compared with those following appendectomy [10,11].

## Conclusion

In the present case, LAMN was diagnosed during macroscopic examination of the appendix and NET was discovered incidentally on histological examination. Therefore, we conclude that appropriate preoperative and postoperative examination of the appendix is the key to the diagnosis of appendiceal tumor.
